# Recurrent synovitis of hip and *MEFV* gene related arthritis in children

**DOI:** 10.1186/s12969-020-00456-3

**Published:** 2020-08-10

**Authors:** Farhad Salehzadeh, Mehrdad Mirzarahimi

**Affiliations:** Pediatric Rheumatology, Pediatric Department, Bouali Children’s Hospital, Ardabil University of Medical Sciences (ARUMS), Ardabil, Iran

**Keywords:** FMF, MEFV gene, Recurrent synovitis of hip

## Abstract

**Background:**

Recurrent and relapsing arthritis has been proposed to describe a group of arthritis with recurring and periodic nature, in which the joints are intermittently involved.

This study reports three non-FMF patients with heterozygous *MEFV* gene mutations and an extraordinary arthritis as a recurrent synovitis of hip (RSH).

**Methods:**

During 16-years from 2003 to 2019 at pediatric rheumatologic clinic among 195 recorded files with chronic oligoarthritis, 3 patients with diagnosis of recurrent synovitis of hip (RSH) were reviewed thoroughly. Peripheral blood was collected from patients and the samples were screened for the 12 common *MEFV* gene pathogenic variants.

**Results:**

This study included three patients, two female and one male with relapsing idiopathic arthritis that has been located on hip joints as a sole manifestation and pathologic findings of *MEFV* mutations as follow: A744S, V726A, and R761H.

**Conclusion:**

On the basis of possible role of *MEFV* gene in different rheumatic disease, *MEFV* gene related arthritis may be considered as a background of RSH particularly in Mediterranean area.

## Background

Recurrent arthritis has been proposed to describe a group of arthritis that has periodic nature, in which the joints are intermittently involved. It may result from genetic, infectious or hereditary and/or acquired background [1]. These include arthropathy of familial Mediterranean fever (FMF) particularly in Mediterranean area; inflammatory bowel disease (IBD) - related arthritis; crystalline arthropathies, such as gout; Whipple’s disease; Behcet’s syndrome; relapsing polychondritis and intermittent hydrarthrosis [2].

Familial Mediterranean fever (FMF) is an autosomal recessive inherited auto inflammatory disorder. It affecting mainly Mediterranean people especially Jews, Armenians, Arabs, and Turks, however; it is now being recognized in other populations, including Iranian, Italians and Greeks.

The clinical manifestations of FMF are characterized by recurrent periods of fever, associated with serositis, erysipelas-like erythema and arthritis [3].

A gene for FMF (*MEFV*) has been identified on the short arm of chromosome 16, and more than 330 mutations in this gene have been demonstrated in patients with FMF [4].

Arthritis is the presenting finding of FMF in some patients, and sometimes it remains as the major manifestation of the disorder [5].

The frequency of arthritis in FMF has been reported to range 40% and or more in different ethnic groups [6]. The most common type of arthritis is large joints self-limited, recurrent, and short lived acute inflammation of the lower extremities [5].

This study reports three non-FMF pediatric patients with heterozygous *MEFV* mutations and an extraordinary arthritis as a recurrent idiopathic and intermittent (left and right) synovitis of hip (RSH), that is not typical for FMF arthritis and known rheumatic disease and discusses about FMF related arthritis and different rheumatic disease that has been affected by *MEFV* gene mutations.

## Material and methods

### Patients

During 16-years from 2003 to 2019 at pediatric rheumatologic clinic among 195 recorded files with chronic oligoarthritis, three patients with diagnosis of idiopathic RSH were reviewed thoroughly and twelve common *MEFV* gene mutations were analyzed to evaluate the role of this mutation as a background of this entity.

Patients had negative histories of FMF manifestations and familial history of FMF. JIA, gout, psoriasis, IBD, SLE, celiac and infectious causes such as brucellosis and some especial disorders such as Ehlers Danlos and joint hypermobility; post-infectious arthritis, pain amplification syndromes and osteochondrosis and palindromic rheumatisim were excluded.

Tel-Hashomer criteria have been used to diagnosis of FMF. None of the patients and their first degree families had FMF related symptoms. Arthritis had been confirmed by imaging including MRI and or ultrasound further than laboratory tests. Patients had taken treatment in short course such as NSAID and Hydoxychloroquine (HCQ) intermittently as DMARDS with suspicion of unclassified and atypical JIA.

The study is complaint with the Helsinki Declaration and was approved by the local Ethics Committee under number (IR.ARUMS.- REC.1396.95). Informed consent was obtained from all the participants, and/or their parents.

### *MEFV* gene mutation analysis

Peripheral blood was collected from patients and the samples were screened for the 12 common pathogenic variants (E148Q, P369S, F479 L, I692del, M680I (G/C), M680I (G/A), M694 V, M694I, K695R, V726A, A 744S, R 761H) by an RDB assay (FMF Strip Assay, Vienna lab, Vienna, Austria) according to manufacturer’s instructions, from fresh blood sampling simultaneously at genetic lab center of the hospital.

## Results

This study included three patients with relapsing and intermittent (right and left) arthritis that has been located on hip joint as a sole manifestation with diagnosis of recurrent synovitis of hip (RSH). The mean attacks was three episodes in year and mean follow up of patients was at least 5 years.

All data of patients and their findings and demographic characteristics are presented in Table 1.

**Table 1:** Patient’s findings and manifestations

Data	Patient 1	Patient2	Patient3
Sex	female	male	female
Age at diagnosis	3.5 yr	4 yr	4.5 yr
Duration of follow-up (years)	4 yr	5 yr	7 yr
Number of episodes per year	3–4	3	3–4
Attack intervals (mean) month	3 to 4 mo	3 mo.	3to 4 mo
Systemic symptoms during attacks	Low grade fever	moderate fever	Low grade fever
Morning stiffness during attacks	No	No	No
*MEFV* Mutations	A744S	V726A	R761H
Average duration of episodes	3 days	3-4 days	5 days
Joint /Right and left	Hip(R/L) intermittently	Hip(R/L) intermittently	Hip(R/L) intermittently

## Discussion

Articular involvement is one of the most common and significant features of FMF. Arthritis usually occurs during recurrences, which can be short-term and self-limiting in nature. It is usually presented as monoarthritis of the lower extremity in large joints. Sometimes arthritis is one of the first symptoms of FMF, and occasionally it is the only episodic symptom of disease in FMF patients [7].

That is usually associated with fever, local redness, swelling and tenderness and positive family history of FMF, *MEFV* mutations analysis, and favorable response to colchicine [5]. Meanwhile 5% of patients have chronic and protracted arthritis, usually of the hips or knees, with symptoms lasting a few months [7].

Chronic involvement of the hip is usually seen as repeated articular or protracted attacks in association with other systemic symptoms; however, chronic osteoarthritis of the hip joint can occur in adult patients without a history of attack at the affected joint [8].

Heller wrote the first comprehensive study about the arthritis in FMF patients, although his work confined to adult patients, he analyzed the clinical, roentgenologic, and histologic features of the articular manifestations of FMF [9].

Eldar studied arthritis in 124 children with well-documented familial Mediterranean fever (FMF), none of this group had arthritis before FMF symptoms, 75% of the patients being under 10 years of age.

Arthritis was noted in the lower extremities 122 (98%), upper extremities17 (14%), and small joints of the hands and feet, 15 (12%).He report three patients with atypical arthritis involving temporomandibular, sacroiliac, and sternoclavicular joints [5]. There was not any patient with exceptional hip involvement and as a sole manifestation of FMF.

Recent findings have shown that *MEFV* gene is associated with other clinical and rheumatologic conditions. Screening of *MEFV* mutations in different rheumatic diseases showed its effective role in these patients with arthritis other than FMF [10].

Several case reports and cohorts suggest an association between FMF and certain inflammatory disorders, such as rheumatoid arthritis [11], JIA [12] systemic onset JIA [13], seronegative spondyloarthropathy [14], Henoch-Schönlein purpura [15], polyarteritis nodosa [16], Behçet’s disease (BD) [17], and Crohn disease [18].

It was shown that *MEFV* mutations in patients with RA were associated with more severe diseases [11]. Furthermore, in Chinese and Indian patients with secondary amyloidosis, the prevalence of p.E148Q mutation was significantly higher than controls [19].

The allele frequencies of *MEFV* mutations were found to be 19.27%, in Turkish children with juvenile idiopathic arthritis which was higher than the general population [12].

Enthesopathy and sacroiliitis occur in some FMF patients, and up to 7.5% of FMF patients are simultaneously diagnosed with Ankylosing Spondyloarthropathy (AS) [20].

Gülhan’s study showed that certain *MEFV* mutations may be acting as susceptibility factors for enthesitis related arthritis (ERA). The presence of two patients with homozygous *M694V* mutations while they lacked other clinical features of FMF at diagnosis of their JIA, also suggests that ERA should be considered in FMF patients [21].

It is a general consensus that spondyloarthropathy in FMF patients is negative for HLA-B27 whilst M694V might be a risk factor for AS [14].

*MEFV* mutations are associated with “subclinical inflammation”; it has been showed by Tunca et al. [22] that, patients who were heterozygous for *MEFV* mutations have increased blood levels of inflammatory markers.

Chetrit reports which defined patients with *MEFV*-related non-FMF entities [10], regarding the *MEFV* gene and its link to such clinical phenotypes may lead to the existence of additional clinical presentations within the auto inflammatory diseases [23].

Furthermore, these *MEFV* gene mutation-associated syndromes will justify a therapeutic trial with colchicine as a main or adjacent drug [10].

These patients diagnosed as idiopathic RSH with relapsing and recurrent arthritis of hip showed *MEFV* gene mutations that include R761H, V726A, and A744S in heterozygote form which are not normal variants of healthy people in this area. The most frequent variants among normal and healthy population are E148Q (18.3%), followed by P369S (3.1%), V726A (2.2%), A744S (1.3%), respectively [24].

Otherwise, in FMF patients of this area followings mutations are the most common variants alleles M694V (20.9%), V726A (12.7%), E148Q (10.7%), M680I (10.3%), M694I (2.1%) [3].

Although transient synovitis of the hip is a common disorder in children, chronic and RSH is a very rare condition. The presence of *MEFV* mutations in patients with RSH and its specific pattern, emphasis the potential role of this gene as a pathogenic background in this condition.

On the basis of possible role of *MEFV* gene in different rheumatic disease RSH could be considered as a *MEFV* gene related arthritis.

Although the outcome of children with one *MEFV* mutation with lack of FMF symptoms continues to be a challenge particularly in pathogenic *MEFV* mutation, periodic clinical visits is recommended during follow up of patients [25].

It is possible that these patients develop FMF related symptoms during their third and or more decades. If it occurs we will confront with a new dilemma; whether RSH is the first presentation of FMF related arthropathy, although it has not been reported up to now, and or it is a separate entity and co-existed with FMF. Because of the limited number of patients in this study a large sample size will be required to confirm this finding, however; RSH is not a common condition in children.

## Conclusion

In our opinion, an idiopathic relapsing periodic arthritis in children with exclusively hip involvement (RSH) is a *MEFV* gene related arthropathy.

## Data Availability

Please contact first author (Salehzadeh F.) for data request.

## Abbreviations

ERA: Entesithis related arthritis
FMF: Familial Mediterranean fever
JIA: Juvenile Idiopathic Arthritis
MEFV: Mediterranean fever Gene
PR: Palindromic Rheumatism
RSH: Recurrent Synovitis of Hip
